# Antimalarial Activity of Axidjiferosides, New β-Galactosylceramides from the African Sponge *Axinyssa djiferi*

**DOI:** 10.3390/md11041304

**Published:** 2013-04-17

**Authors:** Fereshteh Farokhi, Philippe Grellier, Monique Clément, Christos Roussakis, Philippe M. Loiseau, Emilie Genin-Seward, Jean-Michel Kornprobst, Gilles Barnathan, Gaëtane Wielgosz-Collin

**Affiliations:** 1 Faculté des Sciences pharmaceutiques et biologiques, LUNAM Université, Université de Nantes, Groupe Mer, Molécules, Santé-EA 2160, Institut Universitaire Mer et Littoral-FR CNRS 3473, 9 rue Bias, BP 53508, F-44035 Nantes Cedex 01, France; E-Mails: fereshteh.farokhi@univ-nantes.fr (F.F.); emilie.seward@googlemail.com (E.G.-S.); jean-michel.kornprobst@univ-nantes.fr (J.-M.K.); Gaetane.Wielgosz-Collin@univ-nantes.fr (G.W.-C.); 2 Muséum National d’Histoire Naturelle, UMR 7245 CNRS, CP52, 61 rue Buffon, 75231 Paris Cedex 05, France; E-Mail: grellier@mnhn.fr; 3 INSERM CRNA U892, 8 quai Moncousu, BP 70721, F-44007 Nantes Cedex, France; E-Mail: monique.clement@univ-nantes.fr; 4 Faculté des Sciences pharmaceutiques et biologiques, LUNAM Université, Université de Nantes, IICIMED/ERATU-EA 1155 Cancer du Poumon et Cibles moléculaires, 1 rue Gaston Veil, BP 53508, F-44035 Nantes Cedex 01, France; E-Mail: christos.roussakis@univ-nantes.fr; 5 Université Paris-Sud, Faculté de Pharmacie, Chimiothérapie Antiparasitaire, UMR 8076 CNRS, F-92290 Châtenay-Malabry, France; E-Mail: philippe.loiseau@u-psud.fr

**Keywords:** *Axinyssa*, marine sponge glycolipids, glycosphingolipids, antimalarial activity, cytotoxicity

## Abstract

The marine sponge, *Axinyssa djiferi*, collected on mangrove tree roots in Senegal, was investigated for glycolipids. A mixture containing new glycosphingolipids, named axidjiferoside-A, -B and -C, accounted for 0.07% of sponge biomass (dry weight) and for 2.16% of total lipids. It showed a significant antimalarial activity, with a 50% inhibitory concentration (IC_50_) of 0.53 ± 0.2 μM against a chloroquine-resistant strain of *Plasmodium falciparum*. They were identified as homologous β-galactopyranosylceramides composed of 2-amino-(6*E*)-octadec-6-en-1,3,4-triol, and the major one, axidjiferoside-A (around 60%), contained 2-hydroxytetracosanoic acid. Cytotoxicity was studied *in vitro* on human cancer cell lines (multiple myeloma, colorectal adenocarcinoma, glioblastoma and two lung cancer NSCLC-N6 and A549). Results of this investigation showed that axidjiferosides are of interest, because they proved a good antiplasmodial activity, with only a low cytotoxicity against various human cell lines and no significant antitrypanosomal and antileishmanial activity. Thus, it seems that galactosylceramides with a β anomeric configuration may be suitable in searching for new antimalarial drugs.

## Abbreviations

COSY: homonuclear correlation spectroscopy
FAME: fatty acid methyl esters
LCB: long chain base
GC-MS: gas chromatography-mass spectrometry
GL: glycolipid(s)
GSL: glycosphingolipid(s)
amu: atomic mass unit
HMBC: heteronuclear multiple bond coherence
HSQC: heteronuclear single quantum coherence
IC_50_: 50% inhibitory concentration
ESI-MS: electrospray ionization mass spectrometry
TLC: thin layer chromatography
dw: dry weight
MEC: minimum effective concentration

## 1. Introduction

Malaria caused by the Anopheles mosquito-transmitted parasite, *Plasmodium*, is one of the leading causes of mortality and morbidity in more than 100 tropical and subtropical countries of the world, *P. falciparum* remaining the most dangerous species and causing the most lethal form of malaria [1,2]. In spite of some important studies towards the development of an efficient vaccine, there is only a limited number of drugs in widespread use for the treatment of malaria [3,4]. In addition to this paucity of drugs is the rapid development of resistance of this parasite to standard antimalarial drugs, leading to the increasing number of deaths from malaria in sub-Saharan African countries [5,6]. Therefore, the rational development of novel pharmacophores for the purpose of malaria intervention requires the identification of new chemotherapeutic targets [7,8]. The potential of natural products is currently investigated [9]. The new breakthrough in malaria treatment could come with the development of a marine lead compound, taking into account the great potential of marine invertebrates to produce a large array of biological-active metabolites [10,11]. Interestingly, the latter reviews on marine antimalarials published in 2009 did not mention any glycolipid (GL), and it was the same from any other natural sources, including plants [9]. Nevertheless, four new ether diglycosides, named matayosides, were isolated from the Brazilian plant, *Matayba guianensis*, and inhibited the growth of *P. falciparum in vitro* [12]. The administration of the natural killer T (NKT)-cell ligand α-galactosylceramide, KRN 7000, a synthetic compound originally derived from the sponge glycosphingolipids, named agelasphins, resulted in rapid, strong antimalarial activity, inhibiting the development of the intrahepatocytic stages of *P. yoelii* and *P. berghei* [13]. It was the first GL to show the ability of potent antimalarial activity [14,15].

Glycolipids are ubiquitous cell membrane constituents in animals, which play a fundamental role in major phenomena, such as cell-cell recognition and antigenic specificity [16]. They exhibit a wide range of biological functions that might be related to the amphipathic nature of the molecule [15,16]. Several GL, mainly glycosphingolipids (GSL) in particular cerebrosides, have been isolated from a number of marine sources, mainly sponges and echinoderms, and they displayed immunomodulating and antitumor activities [15,16,17,18,19,20,21,22,23,24].

In a previous work on the Senegalese sponge, *Axinyssa djiferi* [25], we isolated a GSL complex mixture, which included nine principal closely related compounds, named axidjiferosides, responsible for an interesting antimalarial activity [26]. In the present work, we wished to confirm the presence of such GSL in this sponge and isolate and identify the main component responsible for the antimalarial activity. Thus, a mixture of only three homologous GSL, named axidjiferoside-A, -B and -C, was isolated from the GSL fraction and displayed an antimalarial activity. The main component, axidjiferoside-A, was characterized by the usual spectroscopic methods. Furthermore, it seemed interesting to study the cytotoxicity and the potential activity of these axidjiferosides on other parasites from the genera *Leishmania* and *Trypanosoma*.

## 2. Results and Discussion

### 2.1. Glycolipid Isolation and Structure Determination

**Figure 1:**
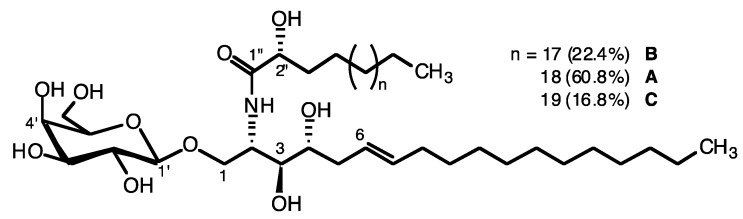
Axidjiferoside-A, B and C, glycosphingolipids from *Axinyssa djiferi*.

The specimens of the Senegalese sponge, *Axinyssa djiferi* [25,26], were extracted with dichloromethane-methanol mixtures, and the crude lipids were fractionated on a silica gel column, eluted with chloroform, acetone (glycolipids) and, then, methanol. Several successive chromatographic steps allowed the isolation of a fraction containing the mixture of three glycosphingolipids (GSL) named axidjiferoside-A, -B and -C. This axidjiferoside mixture accounted for 0.07% of the sponge biomass (dw), 2.16% of total lipids and 17.45% of the total GL and was used for biological studies. The chemical structure and composition of axidjiferosides were obtained by electrospray ionization mass spectrometry (ESI-MS) and NMR studies of the peracetylated GL and by controlled chemical degradation. The peracetylated axidjiferosides exhibited the characteristic signals of a sphingoid base and a β-galactopyranose in the ^1^H-NMR spectrum (Figure 1, Table 1). The ESI-MS showed three molecular ion peaks, corresponding to galactosylceramides. Indeed, the peracetylated major component, axidjiferoside-A, displayed an adduct ion [M + Na]^+^ at *m/z* 1160.7436 (high resolution ESI-MS) in accordance with the formula C_62_H_107_NO_17_Na (a molecular mass of 843.7430 amu (atomic mass unit) for the intact GSL). In addition to the major GSL, two minor GSL (axidjiferoside-B and -C) displayed sodiated molecular ions at *m/z* 1146.7280 and 1174.7593, in accordance with a methylene less or more than the major one.

**Table 1:** ^1^H (500 MHz) and ^13^C (125.75 MHz) NMR data for peracetylated axidjiferosides in CDCl_3_.

Position	δ_H_ ppm, mult., *J* in Hz	δ_C_ ppm
1a	3.88 (dd, *J* = 10.6/3.1)	66.19
1b	3.70 (dd, *J* = 10.6/2.7)	-
2	4.33 (m)	48.09
2-NH	6.81 (d, *J* = 9.0)	-
3	5.12 (m)	71.95
4	4.95 (m)	72.64
5	2.40 (m)	20.75
6	5.50 (dt, *J* = 6.8/15.0)	124.03
7	5.28 (dt, *J* = 7.0/15.0)	134.58
8	1.85 (m)	24.86
terminal methyl	0.87 (t, *J* = 6.8)	19.23
acetates	2.05/2.25/2.17/2.06/2.11/1.99/2.07 (7 s)	169.44/169.86/170.0/170.06/170.18/170.36/170.74
CH_2_ (C9–C17)	1.27 (m)	29.21–30.06
CH_2_ (C4″–C20″)	1.32 (m)	29.21–30.06
1′	4.47 (d, *J* = 7.8)	100.66
2′	5.17 (m)	73.88
3′	5.02 (dd, *J* = 10.4/3.3)	70.79
4′	5.38 (d, *J* = 3.3)	66.96
5′	3.95 (t, *J* = 6.7)	70.79
6′	4.15 (d, *J* = 6.7)	61.06
1″	-	171.08
2″	5.14 (t, *J* = 3.6)	68.6
3″	1.31 (m)	34.41

The structure of axidjiferoside-A, -B and -C was determined on the basis of chemical and spectroscopic evidence. Thus, they contained an unsaturated long-chain amino alcohol as a sphingoid base. All the chemical shifts of the ceramide are given in the ^13^C and ^1^H NMR spectra (Table 1). The sugar linked to the ceramide was identified as galactopyranose by NMR spectroscopy. First of all, the anomeric proton of the β-galactopyranose (δ_H_ = 4.47, d, *J* = 7.8 Hz) was correlated with the anomeric carbon at δ_C_ = 100.66 ppm in the heteronuclear single quantum coherence (HMQC) spectrum. Starting from this proton, all the ^1^H and ^13^C NMR signals of the sugar were assigned by using the homonuclear correlation spectroscopy (COSY), HMQC and heteronuclear multiple bond coherence (HMBC) spectra, and the vicinal proton-proton coupling constants were determined (Table 1). The *galacto* configuration of the sugar, as well as its β anomeric configuration, was established on the basis of the ring proton coupling constants (*J*_1,2_ = 7.8 Hz, *J*_2,3_ = 10.4 Hz, *J*_3,4_ = 3.3 and *J*_4,5_ = 6.7 Hz). The linkage of the galactopyranoside to the ceramide was confirmed by the three bond ^13^C–^1^H couplings of anomeric C-1′ with H-1a and H-1b, observed in the HMBC spectrum. The location of the double bond and its configuration in the dihydrosphingosine moiety was determined from the ^1^H-NMR spectrum in CDCl_3_ with a double triplet at 5.50 ppm (*J* = 15.0, 7.0 Hz) and a double triplet at 5.28 ppm (*J* = 15.0, 6.8 Hz), characterizing an unusual (*E*)-Δ^6^ phytosphingosine. This was supported by two carbon signals at δ 20.75/24.86 for the carbons next to the double bond in the ^13^C-NMR spectrum [21,24], which were assigned by means of COSY and HMQC. COSY correlations were observed between H-5 and H-6 and also H-7 and H-8. Also, the key HMBC correlations from H-5 to C-6, C-7 and C-8 confirmed the location of the double bond. In addition, NMR spectrum showed the presence of terminal methyl groups.

To confirm the structure of the fatty acid methyl esters (FAME) and the long-chain base (LCB), axidjiferosides were subjected to an acid methanolysis. GC-MS analysis of the FAME indicated that the fatty acid moiety was C_22–24_ methyl 2-hydroxylated (mainly C_24_). Indeed, the 2-hydroxy FAME produced the characteristic ions at *m/z* 90 (McLafferty) and *m/z* 103. The results were as follows: 2-OH-23:0, *t_R_ =* 39.6 min (22.4%), *m/z* 384 (M^+^); 2-OH-24:0, *t_R_* = 42.0 min (60.8%), *m/z* 398 (M^+^); 2-OH-25:0, *t_R_ =* 44.3 min (16.8%), *m/z* 412 (M^+^). These fatty acid structures were confirmed by GC-MS analysis of *N*-acyl pyrrolidides showing fragment ions at *m/z* 129 (McLafferty) and the expected corresponding molecular ions. The peracetylated methylglycoside from axidjiferosides analyzed by GC-MS was shown to be methyl galactopyranoside, with a retention time similar to that of an authentic sample. Other diagnostic ions were observed at *m/z* 331 (M–OMe)^+^, 303 (M–OAc)^+^, 243, 200, 157, 145 and 115. The sphingoid base was identified as 2-amino-1,3,4-trihydroxy-octadecene, *t_R_* = 34.12 min, *m/z* 482 (M^+^), and it produced the characteristic ions at *m/z* 43 (base peak) and *m/z* 144. The 2*S*,3*S*,4*R* stereochemistry was assumed by comparison of the ^13^C-NMR chemical shifts of C-2 and C-3 with those of particular GSL, halicerebrosides, amphiceramides and plakosides [18,21,24,27]. The absolute stereochemistry at C-2′ in the 2-hydroxy acyl chains is also assumed to be (*R*), as usually found for glycosylceramides from sponges [16,18,21,24].

In conclusion, axidjiferosides were identified as three homologous β-galactopyranosylceramides composed of 2-amino-(6*E*)-octadec-6-en-1,3,4-triol and the major one, axidjiferoside-A (around 60%), contained 2-hydroxytetracosanoic acid.

### 2.2. Antimalarial, Antileishmanial, Antitrypanosomal and Antiproliferative Activity

The antimalarial activity of axidjiferosides was evaluated against the chloroquine-resistant FcB1/Colombia strain of *Plasmodium falciparum* (50% inhibitory concentration (IC_50_) value for chloroquine of 0.1 μM) [28]. The antiplasmodial activity was determined according to Labaied *et al.* [29]. Axidjiferosides showed a significant activity against *P. falciparum* FcB1, with an IC_50_ of 0.53 ± 0.2 μM.

Axidjiferosides were evaluated *in vitro* for other antiparasitic activities against *Leishmania donovani* (*donovani* forms) and *Trypanosoma brucei* (bloodstream forms) and showed no activity against these parasites (IC_50_ > 100 μM and minimum effective concentration (MEC) > 100 μM, respectively). The antiproliferative activity of axidjiferosides was investigated upon the human cancer cell lines [22,30]. Cytotoxicity and antitumor activity of axidjiferosides, with IC_50_ > 35 μM on two human non-small cell lung cancer cell lines (NSCLC-N6 and A549) and with IC_50_ > 60 μM on three human cancer cell lines (KMS-11, GBM and HCT-116), were non-significant (Table 2).

**Table 2:** 50% inhibitory concentration (IC_50_) and minimum effective concentration (MEC) values (μM) for axidjiferosides.

Plasmodium falciparum FCB1	0.53 ± 0.2
*Leishmania donovani*	IC_50_ > 100
NSCLC-N6	IC_50_ > 35
A 549	IC_50_ > 35
KMS-11	IC_50_ > 60
GBM	IC_50_ > 60
HCT-116	IC_50_ > 60
*Trypanosoma brucei*	MEC > 100

It is of interest to compare the antiproliferative activity of axidjiferosides with those of several GSL having also a single sugar, which is most frequently β-d-glucopyranose, rather than β-d-galactopyranose. The biological activity of such GSL is nowadays little documented. Halicerebrosides from the sponge *Haliclona* sp. are glucosylceramides with a sphingoid base unsaturated at C-6/C-7, like axidjiferosides. Thus, the only notable difference in their structure is the presence of a glucose instead of a galactose. Halicerebrosides displayed a cytotoxic activity against murine leukemia cells, P388 (IC_50_ of 20 μg/mL) [24]. Halicylindrosides from the sponge *Halichondria cylindrata* are GSL containing *N*-acetyl-glucosamine as the sugar head. These GSL showed a cytotoxic activity on leukemia cells, P388, with IC_50_ of 6.8 μg/mL [31]. Furthermore, ophidiacerebrosides isolated from some starfishes showed an antiproliferative activity against murine leukemia cells, L1210, at 2 μg/mL [17]. These GSL contain a β-glucopyranoside as the sugar head and a 9-methyl-branched 4,8,10-triunsaturated long-chain amino alcohol as the sphingoid base. One of them, ophidiacerebroside-C from the starfish *Narcissia canariensis*, displayed an interesting cytotoxic activity on adherent human cancerous cell lines (multiple myelomas, colorectal adenocarcinoma, multiform glioblastoma) with an IC_50_ around 20 μM [23]. Interestingly, the IC_50_ values found for axidjiferosides on the same cancer cells were found to be superior, 60 μM. We could then conceive that the presence of a galactose decreases or cancels the cytotoxicity of the GSL. Unfortunately, data on the cytotoxicity of a galactosylceramide are missing to date. Plakosides A and B, from the sponge *Plakortis simplex*, are β-galactosylceramides possessing a prenylated sugar hydroxyl group and cyclopropane-containing alkyl chains. They are immunosuppressors with a non-cytotoxic mechanism of action [18].

Activity of axidjiferosides against *P. falciparum* was five-times less than those of chloroquine (IC_50_ = 0.10 μM), but, on the other hand, axidjiferosides had no high cytotoxicity effect. In addition, the compound exhibited a specific antimalarial activity, since no activity was observed against kinetoplatids. So, it is of interest for further research work on malaria treatment.

## 3. Experimental Section

### 3.1. General Procedures

High resolution electrospray ionization mass spectrometry (HR-ESI-MS, positive mode, ion-source acceleration 4.5 kV, ion-source temperature 200 °C, methanol as solvent) mass spectra were recorded with a Micromass Zab Spec Tof spectrometer. ^1^H- and ^13^C-NMR, as well as 2D-NMR spectra were obtained on a NMR Bruker Avance-500 spectrometer with triple Probe TBI multinuclear in CDCl_3_ at 500.13 MHz and 125.76 MHz, respectively, with reference to an internal standard of tetramethylsilane. Chemical shifts and coupling constants were expressed in δ (ppm) and Hz respectively. GC-MS spectra were performed on a Hewlett-Packard 5890II gas chromatograph linked to a HP 5989A spectrometer and a HP 98785A integrator, moderately polar column DB-1, 30 m length × 0.25 mm i.d. × 0.33 μm phase thickness. The column temperature was varied, after a pause of 2–4 min from the injection, from 110 to 310 °C, with a slope of 3 °C·min^−1^. Analytical thin layer chromatography (TLC) was performed on precoated silica gel F_254_ plates. After development, the dried plates were sprayed with 50% H_2_SO_4_-vanillin and orcinol reagents. High-performance liquid chromatography (HPLC) was carried out with an Intersphere 5 μm ODS1, C_18_, 250 × 4.6 mm, Interchim, with a 230 nm refractive or UV detector.

### 3.2. Animal Material

**Figure 2:**
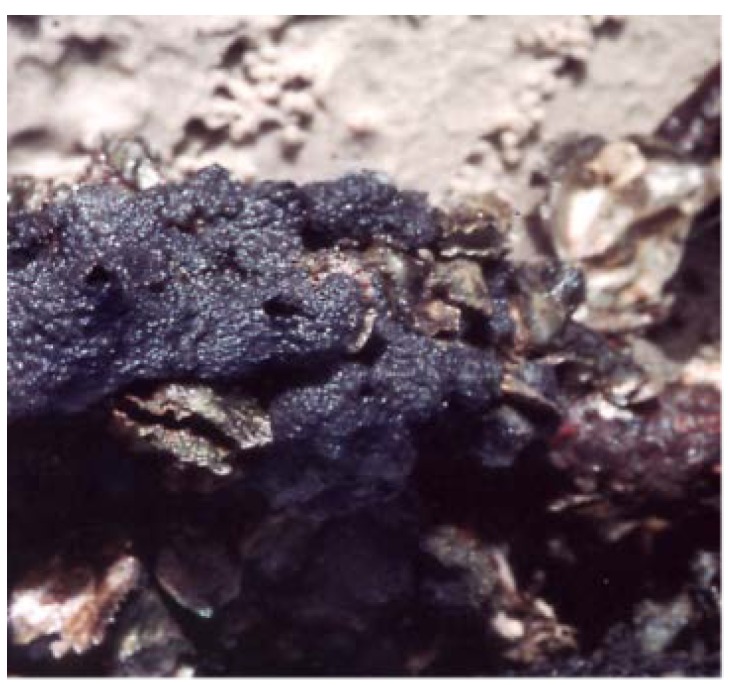
The sponge *Axinyssa djiferi*.

*Axinyssa djiferi* (Figure 2) [25,26] is found along the Senegalese coasts about 150 km south of Dakar, near the village of Djifer, on mangrove tree roots, near the surface at low tide and was collected by hand during an expedition organized by Oceanium of Dakar, on the site named Keur Bamboung (Sine-Saloum). The sponge specimens were frozen shortly after collection and then freeze-dried. A voucher specimen was deposited in the Museum National d’Histoire Naturelle MNHN, Paris, France.

### 3.3. Lipid Extraction and Axidjiferoside Isolation

**Figure 3:**
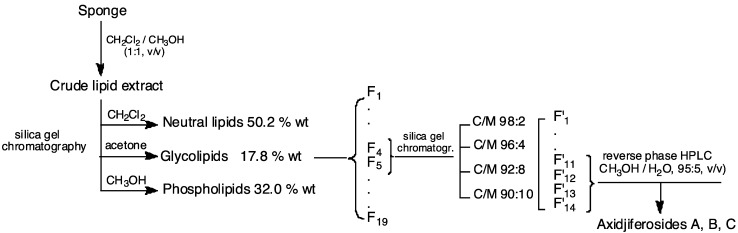
Schematic outline of the experimental protocol for isolation and purification of axidjiferosides.

Whole bodies of the collected specimens were chopped and investigated for their GL fractions. They were twice extracted with CH_2_Cl_2_/MeOH (1:1, v/v) at room temperature. The combined extracts were concentrated *in vacuo* to give the crude extract, which was partitioned between H_2_O and CH_2_Cl_2_. The dried organic layer was concentrated *in vacuo*, and the residue was submitted to a column chromatography over silica gel (230–400 mesh), eluting with pure solvents of increasing polarity, to obtain the different lipid classes: dichloromethane (neutral lipids, 50.2%), acetone (GL, 17.8%) and methanol (phospholipids, 32.0%) (Figure 3).

The last fractions, F′_11_–F′_14_, were pooled and purified by repeated reverse phase HPLC (MeOH/H_2_O, 95:5, v/v) achieved on a 250 × 4.6 mm, Intersphere 5 μm ODS1, Interchim column, until obtaining the fraction containing only axidjiferoside-A, -B and -C. It presented a single spot with a similar polarity to a commercial standard (galactocerebroside with a 2-hydroxy fatty acyl chain type I) (*Rf* = 0.42 on silica gel thin layer chromatography, CH_2_Cl_2_/MeOH/CH_3_CN, 99:1:2, v/v/v).

### 3.4. Acid Methanolysis of Axidjiferosides

An aliquot (3 mg) of axidjiferoside mixture was heated with 1 mL of MeOH/H_2_O/HCl (29:4:3, vol/vol/vol) at 80 °C for 16 h. The reaction mixture was extracted with H_2_O/CH_2_Cl_2_ (3:9, vol/vol). Methyl glycosides were obtained from the aqueous layer, whereas the organic layer contained a mixture of fatty acid methyl esters and the sphingoid base. The FAME were transformed into *N*-acyl pyrrolidides by heating in a pyrrolidine/acetic acid (10:2, vol/vol, 1 mL) during 1 h at 85 °C. The peracetylated methyl glycosides from axidjiferosides (acetic anhydride/dry pyridine, room temperature) were analyzed by GC-MS [column temperature 110 °C (2 min) and then (temperature increasing at 3 °C/min until 240 °C)]; *t_R_* = 37.1 min (methyl galactopyranoside), similar to that of an authentic sample.

### 3.5. Antiplasmodial Activity of Axidjiferosides

According to Labaied *et al.* [29], extracts were prepared in DMSO at a concentration of 10 mg/mL and serially diluted with culture medium before being added to asynchronous parasite cultures (1% parasitemia and 1% final hematocrite) in 96-well microplates. Plates were maintained for 24 h at 37 °C. 0.5 μCi of [^3^H]hypoxanthine was then added to each well, and parasites were maintained for another 24 h. Growth inhibition was determined by comparison of the radioactivity incorporated into the treated culture with that in the control culture maintained on the same plate. Concentrations causing 50% inhibition of parasite growth (IC_50_) were calculated from the drug concentration-response curves. Each experiment was performed in triplicate.

### 3.6. Antileishmanial Activity of Axidjiferosides

Antileishmanial activity was evaluated on the Leishmania *donovani* (MHOM/ET/67/HU3) line, called LV9. Promastigote forms were grown in M-199 medium supplemented with 10% inactivated fetal calf serum, 40 mM HEPES, 100 μM adenosine and 0.5 mg·L^−1^ hemin in the presence of 50 μg·mL^−1^ gentamicin at 26 °C in 5% CO_2_. Peritoneal macrophages were harvested from female CD1 mice three days after an intraperitoneal injection of 1.5 mL of sodium thioglycolate (Biomérieux) and were dispensed into eight-well chamber slides (LabTek Ltd., Melbourn, England) at a density of 5 × 10^4^ cells per well (400 μL per well) in RPMI 1640 medium supplemented with 10% FCS, 25 mM HEPES and 2 mM glutamine. Four hours after, wells were washed to eliminate fibroblasts. After a 24 h incubation period, the macrophages were infected with promastigotes at a stationary phase in a ratio of 10 parasites per macrophage. After 18 h, free promastigotes were eliminated, and intramacrophagic amastigotes were treated with various concentrations of compounds. Pentamidine and amphotericin B were used as reference compounds. The culture medium was renewed 48 h later, and a new culture medium containing the drug was added. The experiment was stopped after five days, and the percentages of infected macrophages were evaluated microscopically after Giemsa staining. IC_50_ values were determined by linear regression analysis. Each experiment was performed in triplicate.

### 3.7. Antitrypanosomal Activity of Axidjiferosides

Antitrypanosomal evaluation was performed as follows: the bloodstream forms of *T. brucei* were purified by centrifugation from the blood of an infected mouse and were maintained *in vitro* for 24 h at 37 °C in a 5% CO_2_ atmosphere in a minimum essential medium supplemented with 25 mM HEPES, Earle’s salts, 2 mM glutamine, 1 g·L^−1^ of glucose, minimum essential medium non-essential amino acids, 0.2 mM 2-mercaptoethanol, 2 mM sodium pyruvate, 0.1 mM hypoxanthine, 0.016 mM thymidine, 15% heat-inactivated horse serum and 50 μg·mL^−1^ of gentamycin. Drug evaluation was carried out in 96-well plates in a final volume of 200 μL containing 2 × 10^5^ trypomastigotes and the compounds to be tested. Pentamidine was used as the reference compound. The minimum effective concentration (MEC) was defined as the minimum concentration at which no viable parasite was observed microscopically. This value was confirmed by injecting intraperitoneally the culture from the well corresponding to the MEC into a mouse to confirm that the non-motile parasites were really killed and not able to divide within mice.

### 3.8. Antiproliferative Activity of Axidjiferosides

The antiproliferative activity of axidjiferosides was investigated upon the cell lines, NSCLC-N6, (derived from a human non-small-cell bronchopulmonary carcinoma, moderately differentiated, rarely keratinized, classified as T2N0M0) [30], A549 (obtained from ATCC collection reference CCL-185) [32], GBM (astrocytoma cells obtained after tumor resection of patients with glioblastoma multiform-primary culture) [33], HCT-116 (colorectal adenocarcinoma cells derived from a patient with Lynch’s syndrome) [34] and KMS-11 (adherent plasma cells obtained from patients with multiple myeloma) [35], as previously described [22,23]. Experiments were performed at least in triplicate, 4 wells per axidjiferoside concentration being used. IC_50_ values were calculated from the dose-response curves.

## 4. Conclusions

The sponge *Axinyssa djiferi*, which lives in the particular biotope of the African mangrove, produced a series of glycosphingolipids, including the three new axidjiferosides isolated in this work. They contained the unusual Δ^6^-phytosphingosine and a not-so-common galactopyranose. Interestingly, axidjiferosides showed a promising antiplasmodial activity, with only a low cytotoxicity against various human cell lines and no significant antitrypanosomal and antileishmanial activity. If α-galactosylceramide, as a NKT cell ligand, showed a rapid and strong antimalarial activity, it seems nevertheless that a galactosylceramide, such as axidjiferoside with a β anomeric configuration, may be suitable in searching for new antimalarial drugs.
